# Neuroregulatory Biomaterials for Intervertebral Disc Regeneration via Neuro-Disc Microenvironment Modulation

**DOI:** 10.34133/research.1062

**Published:** 2026-02-16

**Authors:** Ang Li, Xiaohu Li, Yi Yu, Liang Chen, Juan Wang, Wenguo Cui, Xiaodong Liu

**Affiliations:** ^1^ Department of Orthopedics, Yangpu Hospital, School of Medicine, Tongji University, Shanghai 200090, China.; ^2^ Department of Orthopaedics, Shanghai Key Laboratory for Prevention and Treatment of Bone and Joint Diseases, Shanghai Institute of Traumatology and Orthopaedics Ruijin Hospital Shanghai Jiao Tong University School of Medicine, Shanghai 200025, China.

## Abstract

Intervertebral discs have long been considered to lack neural innervation. However, recent studies have revealed that intervertebral disc degeneration is accompanied by marked pathological nerve invasion. In addition, the transmission mechanism of degeneration and related pain is exacerbated through the neural intervertebral disc microenvironment interaction network. Despite advances in this field, comprehensive reviews remain limited, and systematic analyses are lacking. In this review, we aimed to systematically analyze the potential application of “neural intervertebral disc microenvironment interaction” in the repair of intervertebral disc degeneration. First, in this article, we discussed the neural innervation and functional characteristics of healthy intervertebral discs, elaborated on the mechanism of abnormal nerve growth in the microenvironment of degenerated intervertebral discs, and explained how nerve invasion exacerbates intervertebral disc degeneration and its clinical symptoms by activating pain transmission pathways. Subsequently, we reviewed bioengineering strategies that provided new pathways for the innovative treatment of intervertebral disc degeneration through the synergistic effects of neural regulation and microenvironment interactions. Finally, we explored application prospects of neural-regulated biomaterials in precision medicine, clinical translation, and interdisciplinary fusion, providing a theoretical basis and technical guidance for neural-based intervertebral disc regeneration therapy.

## Introduction

Intervertebral disc degeneration (IDD) is a major cause of low back pain (LBP) [1]. LBP seriously affects the quality of life of patients and imposes a huge burden on the social economy. Globally, more than 800 million people are affected, and both its prevalence and related burden are expected to continue to increase [2]. The occurrence of IDD is closely related to various factors, including mechanical injury, metabolic abnormalities, and persistent inflammatory reactions. These factors contribute to the loss of the intervertebral disc (IVD) structure and function [3]. However, the pathogenesis of IDD remains complex and incompletely understood, particularly the role of nerves in the degenerative microenvironment, which has gradually become a new research focus.

Currently, treatment methods for IDD mainly include conservative treatment and surgical interventions. These methods can alleviate pain and relieve some symptoms; however, they cannot fully restore the complete structure and biological function of the IVD [4]. With the rapid advancement in regenerative medicine, biomaterial-based therapies have provided new avenues for IVD repair. By reconstructing the mechanical support of the fibrous ring, annulus fibrosus (AF), and supplementing the osmotic pressure of the nucleus pulposus (NP), biomaterials can synergistically achieve structural repair and functional recovery of the IVD [5]. These biomaterials not only provide structural support [6] but also can inhibit nerve growth [7], promote organizational regeneration [8], and deliver drugs through intelligent responses, enabling more precise therapeutic effects [9]. However, existing research has mostly focused on mechanical support and component supplementation, with limited exploration of the regulation of the interaction between nerves and the IVD microenvironment.

In recent years, studies have revealed a positive correlation between nerve infiltration density and the progression of IDD [10]. Nerve fibers were once thought to be responsible only for pain transmission; recent evidence, however, suggests that they are involved in maintaining extracellular matrix (ECM) homeostasis in the ECM of IVD [11,12]. Sun et al. [13] have systematically summarized the roles of these nerve fibers and their neurotransmitters in IDD. These nerve fibers act through neurotransmitters, and factors produced in degenerative IVD can reverse the activation of adjacent neurons in the dorsal root ganglion (DRG) and sympathetic ganglia. These signals are then transmitted through the nervous system to the ventromedial hypothalamic nucleus (VMH) and paraventricular nucleus (PVN). In turn, the central nervous system can also release neurotransmitters to IVD via this pathway and directly regulate IVD cell function through corresponding receptors, thereby contributing to the pathological process of IDD. Building on the existing literature, this review further integrates the mechanisms of action of neural, immune, and mechanical networks, and emphasizes the potential strategies of combining biomaterials to achieve precise neural regulation, providing new research perspectives and methodological guidance for IDD intervention. Neural regulation has thus emerged as an important research topic. Interventions targeting the interaction between nerves and the IVD microenvironment, such as local factor delivery, electromagnetic stimulation, and microenvironment reconstruction, have shown potential for relieving pain, promoting tissue regeneration, and inhibiting IDD progression. These strategies not only indirectly regulate the functions of sensory neurons and their associated glial cells that govern IDD but also provide more precise and effective targeted treatment options for IDD.

**Fig. 1.**
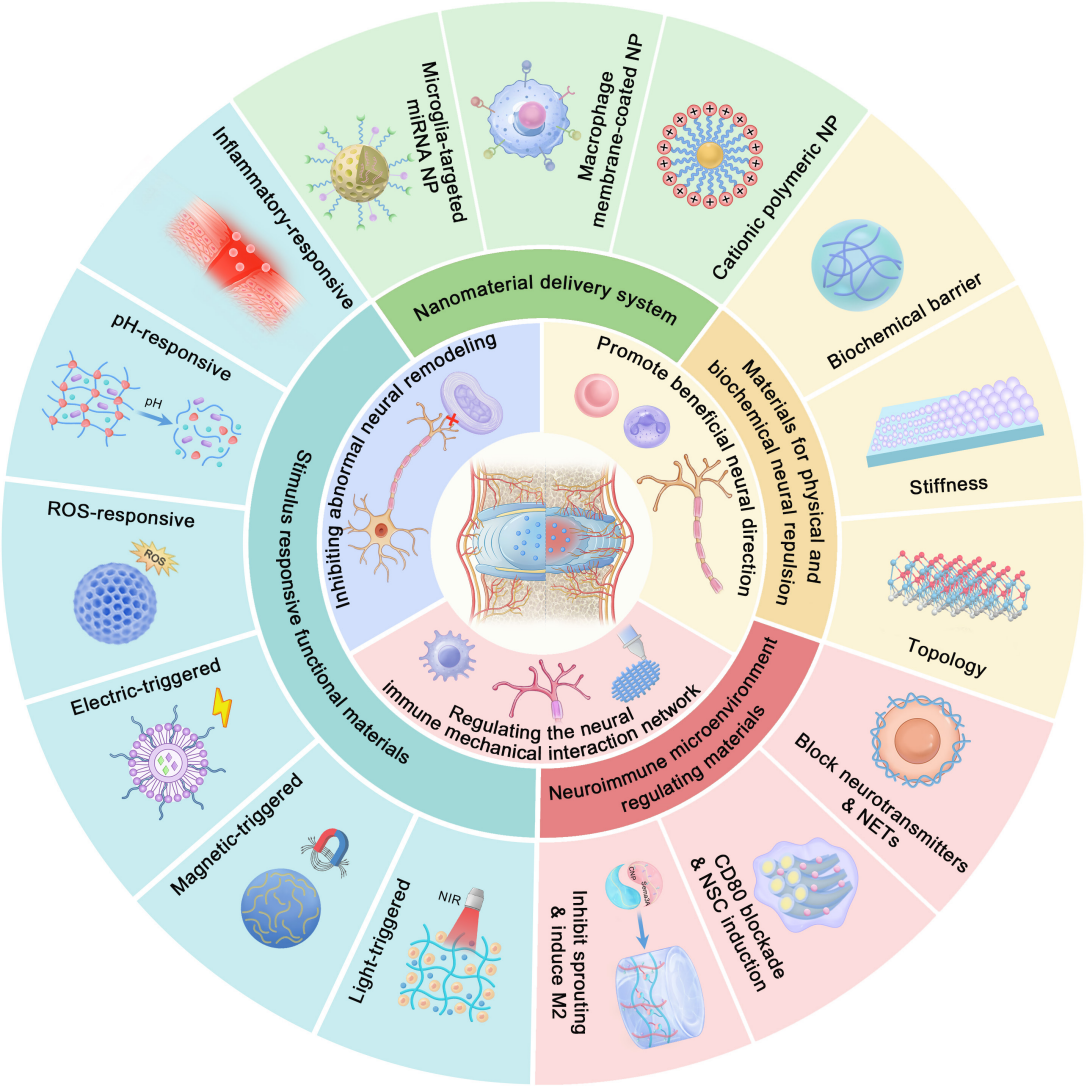
Schematic of collaborative neural regulation and biomaterial strategies for IDD repair.

Therefore, in this study, we aimed to systematically explore the pathological mechanism of neural regulation in IDD and analyze the design concept of neural regulatory biomaterials and their application prospects in IVD repair. The first section introduces the neural mechanisms underlying IDD, with emphasis on the role of nerves. The second part explored the role of neural regulatory strategies in IVD regeneration. Then, the third section discusses the construction and mechanism of the neural regulation of biomaterials. The fourth section discusses application prospects and future research directions for neural regulation in IVD regeneration (Fig. 1).

## Results and Discussion

### The neural mechanism basis of IDD

#### The anatomical structure and neural distribution of IVD

**Fig. 2.**
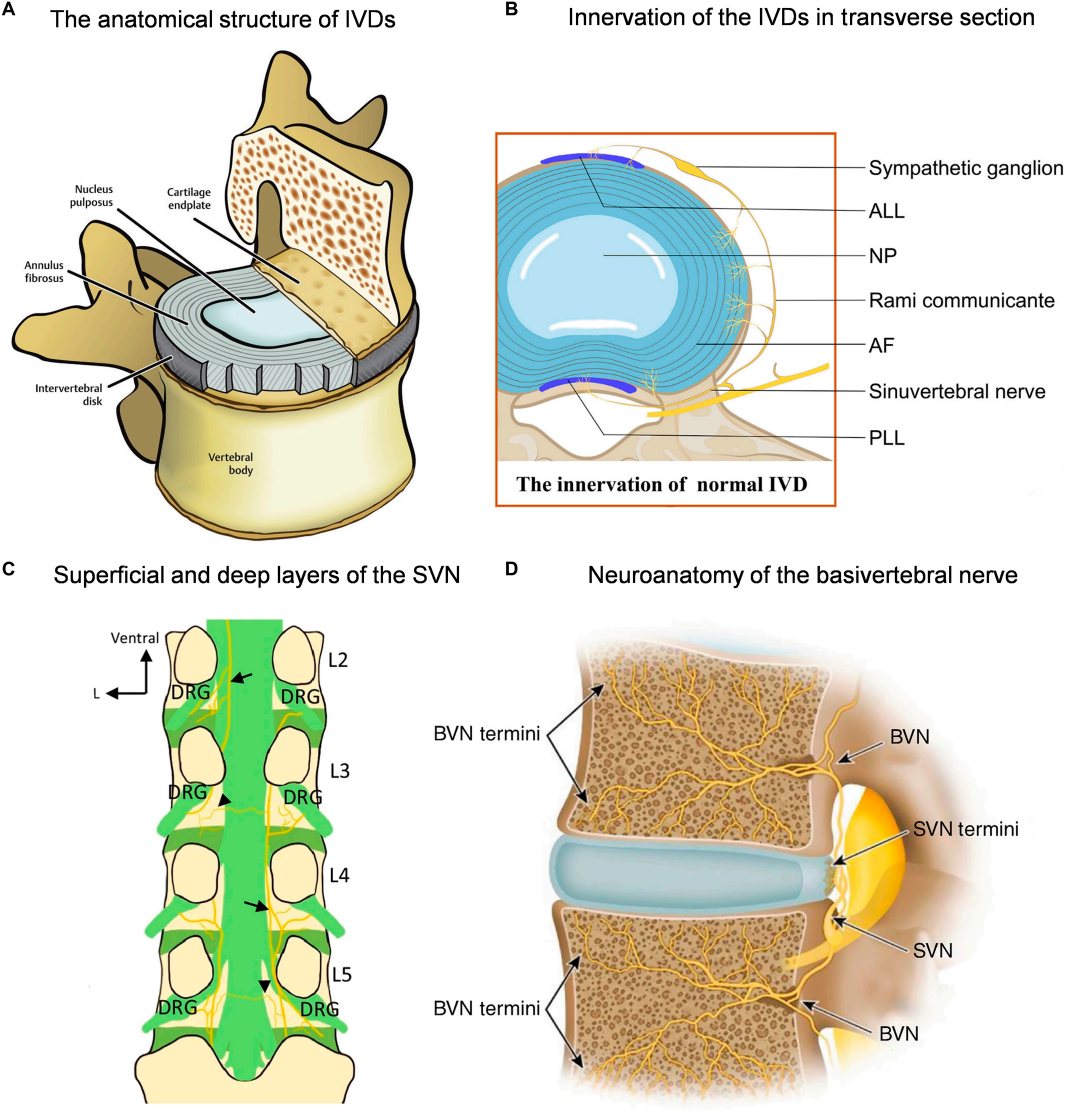
(A) The anatomical structure of IVDs. Reproduced with permission [45]. Copyright 2022, Elsevier. (B) Innervation of the IVDs in transverse section. Reproduced with permission [145]. Copyright 2021, Elsevier. (C) SVN networks, where arrows denote the superficial and arrowheads denote the deep components. Reproduced with permission [146]. Copyright 2025, Springer. (D) Neuroanatomy of the basivertebral nerve. Reproduced with permission [147]. Copyright 2024, Springer.

The IVD is a tough fibrous tissue that connects various vertebral bodies and serves as the main functional unit that constitutes the spinal motion segments [14]. It consists of 3 components: the NP located in the center, the surrounding AF, and the superior and inferior cartilaginous endplates (CEPs) connected to the adjacent vertebrae [15]. NP is a highly hydrated gelatinous tissue mainly composed of type II collagen and proteoglycans, which endows the IVD with the ability to resist compression [16,17]. AF is composed of multiple layers of interlaced collagen fibers, with an outer layer rich in type I collagen and an inner layer enriched with type II collagen and proteoglycans, providing the IVD with multidirectional mechanical strength and structural stability [18]. CEP is a transparent cartilage layer with a thickness of 600 to 1,000 μm, mainly composed of type II collagen, proteoglycans, and water. It functions as a mechanical barrier between the NP and vertebral bone, and a channel for nutrient transport in the IVD [19,20]. Healthy adult IVD is internally avascular and neurogenic, with blood vessels innervating the CEP and outer AF nerves innervating the outer one-third of the AF. Furthermore, nutrients and oxygen passively diffuse into the internal AF and NP (Fig. 2A) [21].

The IVD is a structure with visceral nerve innervation characteristics, which is innervated by both the sympathetic nervous system (SNA) and the sinovertebral nerve (SVN). The anterior part of a normal lumbar IVD is innervated by the SNA, while the posterior part is innervated by the sinovertebral nervous system (Fig. 2B and C) [22]. The SVN is composed of the spinal nerve and the gray communicating branch of the SNA. Its transverse branch innervates the AF, the descending branch innervates the posterior longitudinal ligament and some AF, and the ascending branch gives rise to the basivertebral nerve near the pedicle [23]. The basivertebral nerve and vertebral blood vessels pass through the intervertebral foramen, enter the center of the vertebral body to form a cluster of nerve vessels, and branch out to supply the CEP to the upper and lower vertebral bodies (Fig. 2D) [24]. In the normal NP, there was no nerve growth. In contrast, 3 types of nerve elements were detected in the CEP and AF, including sensory nerve fibers, independent of blood vessels, perivascular nerve fibers, and mechanoreceptors [25]. This neural distribution constitutes a complex neural IVD microenvironment interaction network, which plays a role in regulating the physiological and pathological processes of IVD.

##### Sensory nerve fiber innervation in IVD

Sensory nerves are responsible for transmitting stimulus signals, including sensations such as pain and pressure [26]. In the IVD, these nerves are primarily composed of myelinated A-δ fibers and unmyelinated C fibers [22], which synthesize neuropeptides involved in nociception, such as calcitonin gene-related peptide (CGRP) and substance P (SP) [27]. CGRP is widely regarded as a pain-related neuropeptide associated with nuclear factor κB (NF-κB) in the IDD process [28], but some studies have also suggested that it can promote chondroitin sulfate synthesis by enhancing the expression of chondroitin sulfate synthase 1 (CHSY1), thereby maintaining the ECM homeostasis of NP [11]. Similarly, while SP is generally involved in the transmission of pain and inflammation in IVD [29], low-dose SP can promote the proliferation of AF cells and the deposition of proteoglycans [30].

Mechanical receptors are key components of sensory nerve endings and are widely distributed in the superficial and longitudinal ligaments of the AF, especially in the anterior part of the L5–S1 IVD [31]. The mechanoreceptors located at nerve endings can perceive external mechanical stimuli such as pressure, tension, and compression and convert them into biochemical signals that regulate the synthesis and degradation of the ECM in the IVD microenvironment [32]. In degenerative IVD, the number and activity of mechanoreceptors usually increase, which may not only alter the cell’s response to mechanical stress but also promote the occurrence of degenerative pain through abnormal signal transduction [33]. Therefore, mechanoreceptors play a dual role in maintaining the mechanical stability of IVD and regulating the metabolic balance, and their abnormal expression is an important pathological feature in the process of IDD.

Anatomically, the sensory nerve innervation of the IVD exhibits distinct segmental and intersecting features. Spinal nerve innervation is conically distributed, with one spinal nerve innervating multiple IVDs, while a single IVD is regulated by multiple segments and spinal nerve levels [34]. The labeling experiment in a rat model also showed that the sensory input of the L5–L6 IVD was primarily concentrated in the L1–L3 segment, particularly in the L2 DRG, indicating noncorresponding segmental cross-segmental projection features [35]. This convergence and cross-dominance form a complex sensory input network that provides an anatomical basis for IDD-related pain.

##### Sympathetic nerve fiber innervation in IVD

Compared to sensory nerves, sympathetic nerves play an important role in maintaining metabolic homeostasis and rhythmic regulation of IVD tissues, although they do not directly mediate pain transmission. The SNA that controls the IVD mainly originates from the paraspinal ganglia and reaches the periphery of the IVD through the posterior branch of the spinal nerve [36]. The sympathetic nerve fibers usually do not directly enter the interior of the IVD but instead achieve critical interactive communication through the SVN [37]. The SNA has a wide impact on the proliferation and differentiation of IVD cells by secreting various neurotransmitters such as norepinephrine (NE), neuropeptide Y (NPY), and vasoactive peptide (VIP) [38]. Among them, NE generally works by activating the ERK pathway [39], which plays a key role in IVD matrix degradation [40]. NPY exhibits the ability to inhibit ECM degeneration of NP in vitro, suggesting its protective role in regulating homeostasis [41], while VIP delays IDD progression via the fibroblast growth factor receptor 2 (FGFR2)/FGF receptor substrate (FRS)/AKT signaling pathway [42].

The SNA exhibits rhythmic activity that is regulated both centrally, primarily by the brainstem, and locally by the spinal cord [43]. The tension level and neurotransmitter secretion of the SNA show marked fluctuations under the influence of circadian rhythms. Disruption of the circadian rhythm can lead to sustained activation of the SNA and an abnormal increase in neurotransmitters such as NE. This, in turn, triggers sustained activation of proinflammatory and matrix degradation signals in IVD cells, ultimately accelerating IDD [44]. Therefore, rhythmicity of the SNA is a fundamental characteristic of the nervous system and plays an important role in the regulation of the IVD microenvironment and degenerative pathology.

#### Neurological factors are involved in the progression of IDD

As age increases or with exposure to risk factors, such as obesity and smoking, the ECM of IVD cells is degraded. This leads to the loss of NP hydration and a decrease in overall IVD height and stiffness, ultimately resulting in IDD [45]. As degeneration progresses, the CEP undergoes calcification and microporous occlusion, which reduces the transport efficiency of essential nutrients to the IVD [46]. To enhance the internal nutrient supply, blood vessels and nerves grow toward the NP and internal AF [47]. On the other hand, IDD causes cracks in CEP and AF, which allow for communication with the external environment and allow immune cells to enter the IVD [48], disrupting the intact structure of healthy IVDs and further promoting neural ingrowth [49]. Collectively, these series of changes provide an important foundation for the involvement of neural factors in IDD.

Abnormal growth of nerve fibers not only induces IVD-induced pain [50] but also promotes IDD progression [51]. The nervous system interacts with the IVD microenvironment through multiple mechanisms, such as inflammation activation, neurotrophic factor expression, nerve regeneration, and immune regulation. These processes highlight the core role of the neuro-IVD microenvironment interaction in the progression of degeneration. A bidirectional signaling pathway of inflammatory and neurotrophic factors is formed between peripheral sensory neurons, the central nervous system, and peripheral glial cells, constructing a feedback amplification neural immune metabolic network. This network maintains the continuous transmission of pain signals but also drives local matrix degradation and tissue structure remodeling, accelerating the IDD process.

##### Inflammatory neural regeneration pathway

Toll-like receptor 4 is expressed on the surface of IVD cells, which can recognize exogenous stimuli and release inflammatory factors, including tumor necrosis factor-α (TNF-α), interleukin-1β (IL-1β), and IL-6 [52]. As degeneration progresses, rupture of the AF breaks down the original barrier, leading to immune cell infiltration into the NP region [53]. Among these, mast cells are one of the earliest infiltrating cells in IVD lesions [54]. Through degranulation, they release histamine and bioactive substances, promoting nerve growth and inflammatory cascade reactions [55]. Macrophages have also been widely observed in human and mouse IDD models [56,57]. They secrete various inflammatory factors [58] and promote the expression of nerve growth factor (NGF) and CGRP through autocrine or paracrine mechanisms [59], thereby exacerbating inflammation and degeneration [60]. As the disease progresses, local tissues evolve from acute inflammation to a chronic state, with macrophages showing an M2 polarization tendency. This promotes the formation of new capillaries, and the endothelial cells of neovascularization secrete neurotrophic factors such as NGF, which in turn induce the growth of nerve fibers [61]. In addition, lymphocytes have been identified as the key mediators of acquired immune responses in degenerated IVD tissues [62,63]. Activated lymphocytes can release various inflammatory factors that participate in local immune inflammatory responses and may also up-regulate the expression of neurotrophic factors or mediate neural activation [64]. The inflammation-mediated neural regeneration process reflects the complex interaction between multiple types of cells and signaling pathways in degenerative IVD, promoting the establishment and maintenance of the “neuro-IVD microenvironment interaction”.

**Fig. 3.**
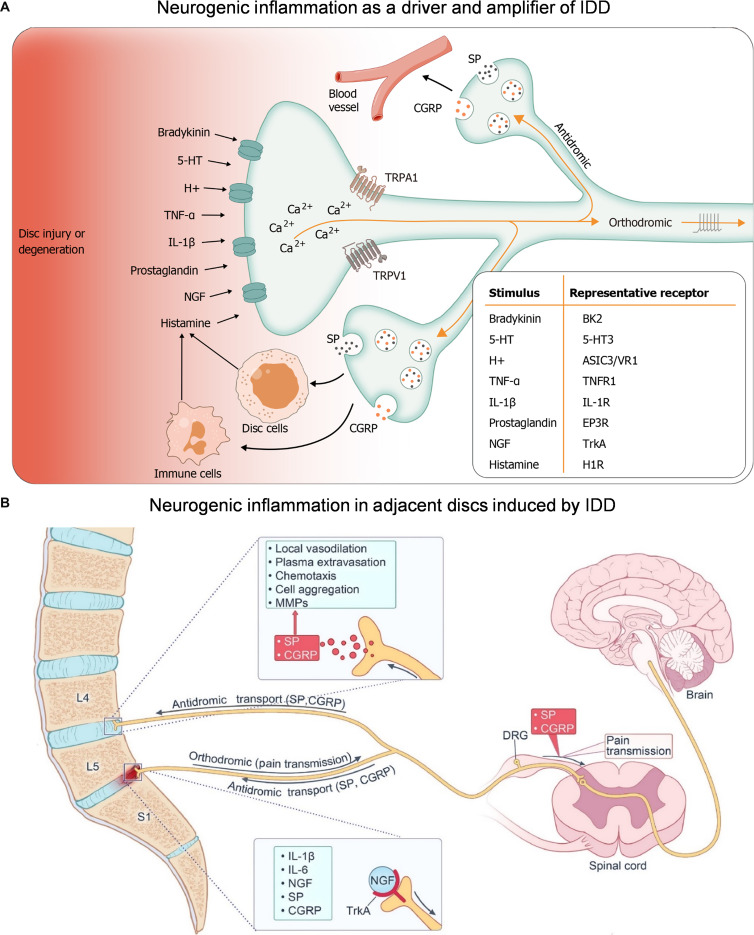
(A) Neurogenic inflammation as a driver and amplifier of IDD. IDD triggers neurogenic inflammation via nociceptor activation and neuropeptide release (SP and CGRP), promoting a self-amplifying loop of inflammatory mediator release from neuronal and non-neuronal cells. Reproduced with permission [67]. Copyright 2025, Baishideng Publishing Group. (B) Neurogenic inflammation in adjacent discs induced by IDD. Neurogenic inflammation initiated by L5–S1 degeneration spreads to adjacent discs via antidromic transport of SP and CGRP. Reproduced with permission [68]. Copyright 2024, Elsevier.

Notably, sensory nerves can also be active drivers of inflammatory responses. Neurogenic inflammation refers to a local inflammatory response triggered by the release of neuropeptides (such as SP and CGRP) from sensory nerve endings at nonsynaptic sites. These neuropeptides promote vasodilation, increased vascular permeability, and immune cell recruitment [65]. After an IVD injury, the sensory neurons that control the IVD can continuously release neuropeptides. In turn, this induces local vasodilation and immune cell recruitment, and activates IVD cells to release proinflammatory cytokines and neurotrophic factors [66]. This atypical inflammatory pattern, starting from sensory nerve endings, is called IVD neurogenic inflammation (Fig. 3A) [67].

In addition, neurogenic inflammation is not limited to the primary damaged area. After inducing IVD injury in a rat segment, the levels of neurotransmitters in adjacent healthy segments were markedly increased, accompanied by the synchronous up-regulation of inflammatory cytokine levels (Fig. 3B)​​ [68]. This suggests that neurogenic inflammation may play a key role in the multi-segmental expansion of IDD and serves as an important mediator of nerve regeneration and pathological remodeling.

##### Neurotrophic factors and pain pathways

Inflammatory factors such as IL-1β and TNF-α directly act on pain receptors, reducing the excitation threshold of peripheral neurons and enhancing their sensitivity to mechanical and chemical stimuli. In addition, they drive NP and AF cells to secrete neurotrophic factors [including nerve growth factor (NGF) and brain-derived neurotrophic factor (BDNF) [69]], which attract sensory nerve endings to invade the inner layer and promote the formation of “neuro–IVD microenvironment interaction” [70]. In turn, inflammatory mediators themselves can directly activate pain pathways, making it easier for neurons to trigger pain impulses [71]. On this basis, SP and CGRP released by sensory neurons form a positive feedback loop through vasodilation and immune cell recruitment, and exacerbate the inflammatory response and pain experienced during IVD [72].

Notably, the ability of neurons to perceive changes in the microenvironment also plays a key role in pain signal transduction. Transient receptor potential (TRP) channels are key ion receptors widely present in the nervous system and participate in environmental stimulus perception and neural signaling regulation [73]. TRPV1-positive sensory neurons that dominate the IVD mostly coexpress CGRP and release this neuropeptide upon nociceptive stimulation [74,75]. Additionally, TRPV1 is sensitive to acidic pH and inflammatory factors, and hence plays a key role in IDD-related pain [76,77]. TRPV2 was initially considered a thermosensitive channel [78]; however, its expression is up-regulated in the DRG and promotes the release of CGRP under inflammatory conditions [79,80]. Furthermore, TRPA1 is widely distributed in nociceptive sensory neurons. Upon activation, it can release neuropeptides and induce edema and leukocyte infiltration [81,82].

Pathological stimulation of the IVD affects peripheral neurons and activates central and peripheral glial cells. In a mouse model of IDD, astrocytes and microglia in the spinal dorsal horn are robustly activated [83,84]. Clinical studies have revealed that the level of the glial cell activation marker translocator protein (TSPO) is elevated in the brains of patients with chronic LBP, suggesting that central glial cell activation plays an important role in IVD-induced pain [85]. In peripheral glial cells, the IVD puncture model up-regulated satellite glial cell expression in the corresponding DRG [86]. These activated satellite glial cells can enhance the excitability of DRG neurons, thereby intensifying peripheral sensitization and sustaining chronic pain [87]. Therefore, synergistic activation of central and peripheral glial cells plays a central role in IVD-induced pain.

**Fig. 4.**
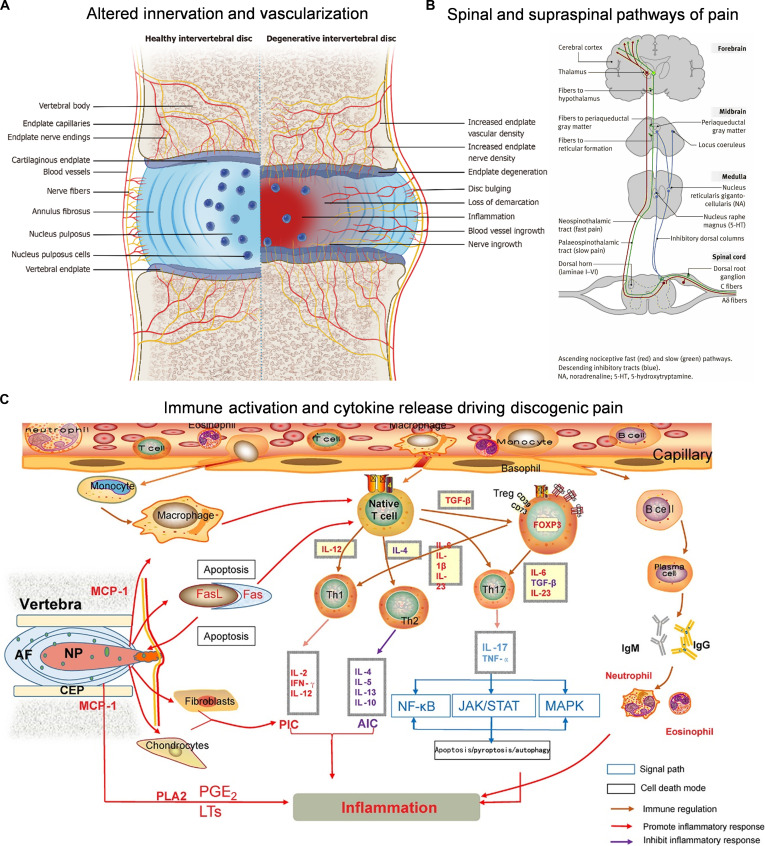
(A) Altered innervation and vascularization in Degenerative IVDs. In healthy discs, nerves and vessels are confined to the outer annulus and endplates. Degeneration leads to AF-NP boundary loss, NP cell reduction, and pathological ingrowth of nerves and vessels into the inner disc and endplate. Reproduced with permission [67]. Copyright 2025, Baishideng Publishing Group. (B) Primary afferent nerve fibers, with cell bodies in the dorsal root or trigeminal ganglia, terminate in the spinal dorsal horn via varying routes. Reproduced with permission [50]. Copyright 2009, Elsevier. (C) Immune activation and cytokine release driving discogenic pain. NP exposure activates immune cells and cytokines (PICs/AICs), which sensitize sensory neurons and drive pain. Reproduced with permission [148]. Copyright 2022, Wiley.

The generation of pain signals begins with the activation of peripheral nociceptors, where A-δ and C-type fibers transmit stimulation signals to the dorsal horn of the spinal cord and relay them via ascending pathway to the thalamus and cerebral cortex, resulting in pain perception (Fig. 4B) [50]. However, harmful stimuli to the IVD can also cause pain in the lower back and limbs [88]. The involvement of pain in the L2 corticospinal area is generally caused by pathological stimulation of the L5S1 IVD, and its transmission pathways are the IVD, SVN, L5 sympathetic ganglia, L2 sympathetic ganglia, white communicating branch, L2 DRG, L2 spinal cord, and cerebral cortex [89]. In rats, the inguinal skin and the sympathetic afferent sensation of the L5L6 IVD project simultaneously to the DRG of the L1 and L2 spinal nerves; thus, the pathological stimulation of this IVD can cause referred pain in the inguinal region [90]. Similarly, testicular pain is generally caused by stimulation from L4L5 IVD lesions, and its conduction pathway is the IVD, which converges with the reproductive femoral nerve and iliac inguinal nerve reproductive branch dermatome through the L1 and L2 DRGs [91].

##### Neural innervation remodeling and chronic pain formation

In normal IVD, nerve fibers are confined in the outer layer of the AF [92], where both physical barriers (Aggrecan) and chemical repellents (Semaphorin-3A) maintain the IVD structure [93,94]. However, during IDD, elevated levels of inflammatory and neurotrophic factors disrupt this balance and induce the inward growth of nerve fibers (Fig. 4A) [95–97]. The sensory nerve fibers that grow into the IVD secrete neuropeptides, including CGRP and SP, which are recognized by IVD cells. They locally infiltrate immune cells and induce them to secrete more NGFs and neuropeptides, further enhancing neural structural remodeling [71]. On this basis, NGFs enhance synaptic transmission and neural sensitivity, amplify pain signals, and maintain chronic states (Fig. 4C) [98]. In addition, adverse microenvironments such as acidity and inflammation in IDD lead to the depolarization of peripheral nociceptive nerve endings [99]. The resulting electrical signals are transmitted to the brain through nociceptive neurons, somatic DRGs, and the spinal cord sensory gray matter dorsal horn [100], ultimately inducing neuroplasticity and pain sensitization. This enhances central response to pain signals and promotes the maintenance of chronic pain states [99]. Consequently, the process is a manifestation of the continuous evolution and projection of the “neuro-IVD microenvironment interactions” to the central level.

### The role of neural regulatory strategies in inhibiting IDD

Neuromodulation inhibition of IDD is an emerging therapeutic strategy aimed at intervening in the “neuro-IVD microenvironment interaction” process to reverse the degeneration process and restore tissue homeostasis. IDD is often accompanied by abnormal neural growth, chronic inflammation, and matrix degradation. Beyond serving as a conduit for pain transmission, the nervous system actively contributes to degenerative pathology by releasing neurotrophic factors, regulating immune responses, and affecting cell metabolism.

#### Promote beneficial neural direction

**Fig. 5.**
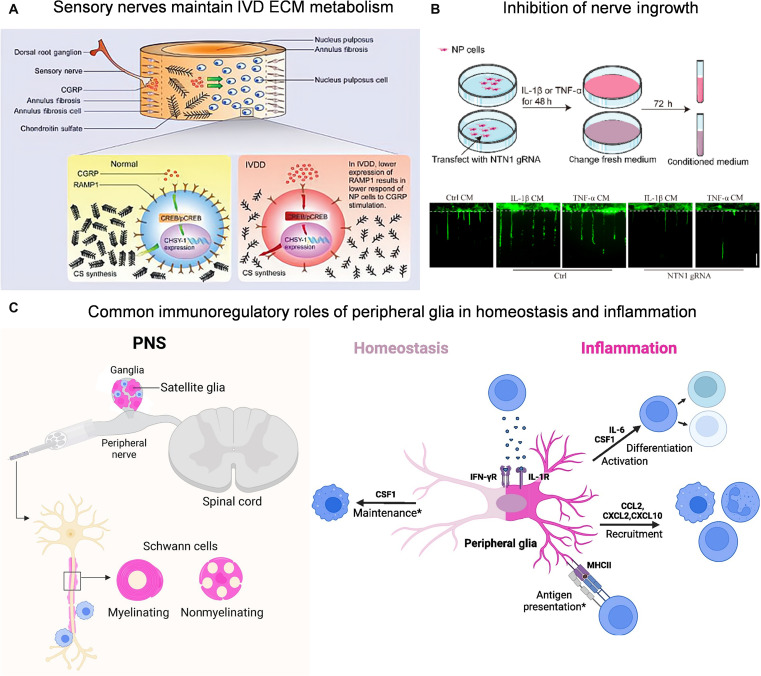
(A) Sensory nerves maintain IVD ECM metabolism through the CGRP/CHSY1 axis. Reproduced with permission [11]. Copyright 2022, Wiley. (B) Netrin-1 was knocked out using CRISPR-Cas9 to inhibit nerve ingrowth. Reproduced with permission [109]. Copyright 2023, Elsevier. (C) Peripheral glia modulate immune cell activity in both homeostasis and inflammation, linking immune responses to neuronal signaling and contributing to neuro-immune crosstalk. Reproduced with permission [118]. Copyright 2025, Elsevier.

The sensory nerves surrounding the IVD not only are involved in pain transmission but also directly regulate the matrix metabolism of IVD cells by releasing neuropeptides (such as CGRP). This regulation helps to the level of water-containing macromolecules such as chondroitin sulfate in the NP. Conversely, blocking sensory nerves leads to a marked decrease in glycosaminoglycan content in the IVD, indicating the importance of neuronal activity for maintaining matrix integrity (Fig. 5A) [11].​ In addition, bidirectional regulation exists between the sensory system within the IVD and the central nervous network. Targeting the sensory mechanism within the IVD can inhibit the degeneration process and also alleviate related pain symptoms [101]. Among these internal sensory pathways, prostaglandin E₂ (PGE₂) exhibits a dose-dependent dual effect. At physiological concentrations, it maintains local homeostasis by activating prostaglandin E (EP) receptors within the IVD. However, when it abnormally increases, it can induce degeneration-related signals [102]. Moderate sensory nerve activity and neurotrophic factor signaling provide not only the necessary physiological conditions for IVD structural repair and cellular function but also a theoretical foundation for neural regeneration strategies.

#### Inhibiting abnormal neural remodeling

The prevention of pathological nerve hyperplasia is essential for IVD regeneration and repair. First, the highly negatively charged physical barrier can be restored by supplementing or reconstructing the key component aggrecan, thereby hindering the invasion of sensory nerve fibers into the inner layer [103]. At the same time, targeting abnormal neurotrophic factor signals is also a key strategy. For instance, anti-NGF antibodies can inhibit nerve fiber proliferation induced by NGF secreted by IVD cells [104], while TRPA1 inhibitors can reduce the expression of CGRP in DRG neurons, indirectly alleviating nerve sensitization [105]. In addition, activation of inflammatory signaling pathways is an important source of neural remodeling abnormalities. Inhibiting the NF-κB pathway or targeting key proinflammatory factors such as IL-1β can effectively reduce the up-regulation of neurotrophic factors and abnormal invasion of nerve fibers [106,107].

In addition to anti-inflammatory approaches and channel blockade, direct intervention in the release mechanism of neuropeptides has become a cutting-edge direction in neuroimmune interventions. Studies have shown that blocking SNARE (soluble N-ethylmaleimide-sensitive factor attachment protein receptor)-mediated vesicle release in neurons can effectively reduce neuropeptide release [108]. In addition, chemical barriers can be strengthened by regulating axonal guiding molecules. In a rat model, Netrin-1 inhibitors markedly reduce neurogenesis and angiogenesis in degenerative IVD and alleviate pain sensitization (Fig. 5B) [109]. However, restoring the expression of Semaphorin-3A in the outer layer of healthy AF can inhibit nerve invasion [110]. Furthermore, the vitamin B6 analogs pyridoxine (Pyr) and vincristine (Vcr) can induce DRG axonal contractions without obvious cytotoxicity, indicating promising application prospects as local neuroinhibitory factors [111]. The above multiple strategies aim to maintain the normal structure and function of IVD while effectively suppressing the formation of pathological pain neural networks and interrupting the pathological circuit of “neuro-IVD microenvironment interaction” evolving toward chronicity.

#### Regulating the neural immune mechanical interaction network

Healthy NPs are similar to the vitreous body of the eye and have a certain degree of immunity; however, an abnormal mechanical load can damage their physical barrier, leading to macrophage infiltration [112]. The inflammatory factors secreted by these macrophages promote the production of neurotrophic factors, such as NGF, thereby inducing the invasion of sensory nerve fibers and activating pain pathways [113]. In addition to the sensory nerves, glial cells play an important role in immune regulation [114,115]. Mechanical stress can be transmitted along nerve axons to the central nervous system, activating microglia and forming a sustained neurogenic inflammatory microenvironment [116]. Peripheral immune and central glial cells interact bidirectionally, driving neuroinflammation and amplifying chronic pain and IDD progression [117]. Similarly, peripheral glial cells can participate in inflammatory responses and maintain tissue homeostasis by regulating immune cell activity, revealing their undeniable immune regulatory role in the neural–immune interaction network [118]. Therefore, accurately intervening in the key interaction links in this network is becoming a new strategy for the systematic treatment of IDD (Fig. 5C and Table 1).

**Table 1.** The mechanism of neural regulation in IDD inhibition

Category	Representative factors	Typical interventions	Ref.
Modulate beneficial neural signaling	CGRP, CHSY1	Enhance sensory neuroanabolic signaling	[11]
	PGE_2_-EP receptor	Regulating EP receptor signaling	[101,102]
Suppress pathological nerve remodeling	Aggrecan	Replenish ECM barriers against nerve invasion	[103]
	NGF, TRPA1, NF-κB, IL-1β	Inhibiting abnormal neurotrophic factor signals	[104–107]
	SNARE	Block neuropeptide release	[108]
	Netrin-1, Semaphorin-3A, Pyr, Vcr	Inhibit axon guidance cues for aberrant innervation	[109–111]
Regulate neuro-immune-mechanical interactions	Mechanical stress, macrophages, glial cells	Modulate neuro-immune crosstalk; control glial and macrophage activation; alleviate stress-induced inflammation	[112–118]

CGRP, calcitonin gene-related peptide; CHSY1, chondroitin sulfate synthase 1; PGE_2_, prostaglandin E_2_; EP receptor, prostaglandin E receptor; ECM, extracellular matrix; NGF, nerve growth factor; TRPA1, transient receptor potential ankyrin 1; IL-1β, interleukin-1β; SNARE, soluble N-ethylmaleimide-sensitive factor attachment protein receptor; Vcr, vincristine; Pyr, pyridoxine

### Regulating the neural immune mechanical interaction network

As the key role of neural mechanisms in the pathogenesis and pain maintenance of IDD has become increasingly clear, precise intervention of neural activity through material means has become an important direction in regenerative medicine research. Biomaterials can not only serve as delivery platforms for drugs, genes, and cells but also achieve multidimensional regulation of sensory neurons, glial cells, and even neuroimmune networks. These effects can be achieved by responding to internal microenvironment changes, external physical field stimuli, or synergistic effects with the immune system, providing a realistic path for intervening in the “neuro-IVD microenvironment interaction”.

#### Nanomaterial delivery system

**Fig. 6.**
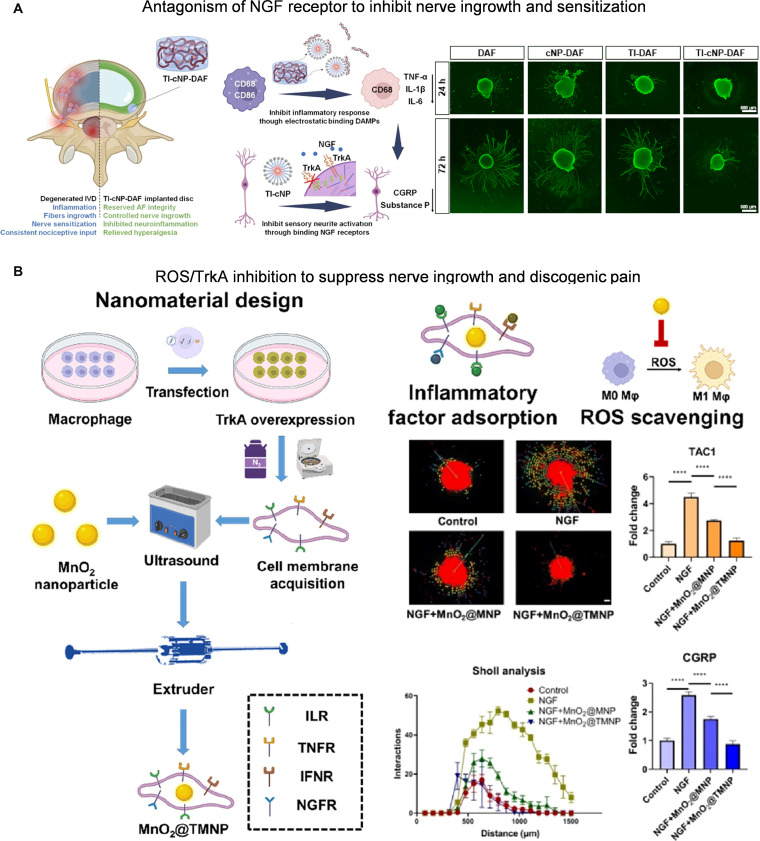
(A) A composite hydrogel delivering TrkA-IN-1 inhibits NGF-induced nerve ingrowth and sensitization in degenerative IVDs. Reproduced with permission [119]. Copyright 2023, Elsevier. (B) Delivery of MnO_2_@TMNP suppresses NGF-mediated nerve ingrowth by scavenging ROS and sequestering inflammatory mediators. Reproduced with permission [120]. Copyright 2022, American Chemical Society.

Using nanomaterials as carriers, drugs, genes, or cells can be delivered to targeted IVD nerves. For example, by loading the NGF receptor antagonist tropomyosin receptor kinase A (TrkA)-IN-1 into the ionic nanopolymer and embedding it within the acellular chemical fiber-ring matrix hydrogel, the composite system could be sustained in the IVD, effectively inhibiting excessive growth of nerve fibers and the production of pain mediators (Fig. 6A) [119]. Similarly, MnO₂ nanoparticles wrapped in macrophage membranes can be delivered to degenerative IVD disease and down-regulate the expression of pain neuropeptides CGRP and SP in the DRG, effectively alleviating discogenic pain (Fig. 6B) [120]. For gene delivery, nanoparticles with the surface-modified microglial-targeting peptide MG1 were used to precisely deliver miR-26a-5p to microglia and alleviate various types of chronic pain for 6 weeks via the nonclassical Wnt5a pathway [121].

#### Stimulus-responsive functional materials

**Fig. 7.**
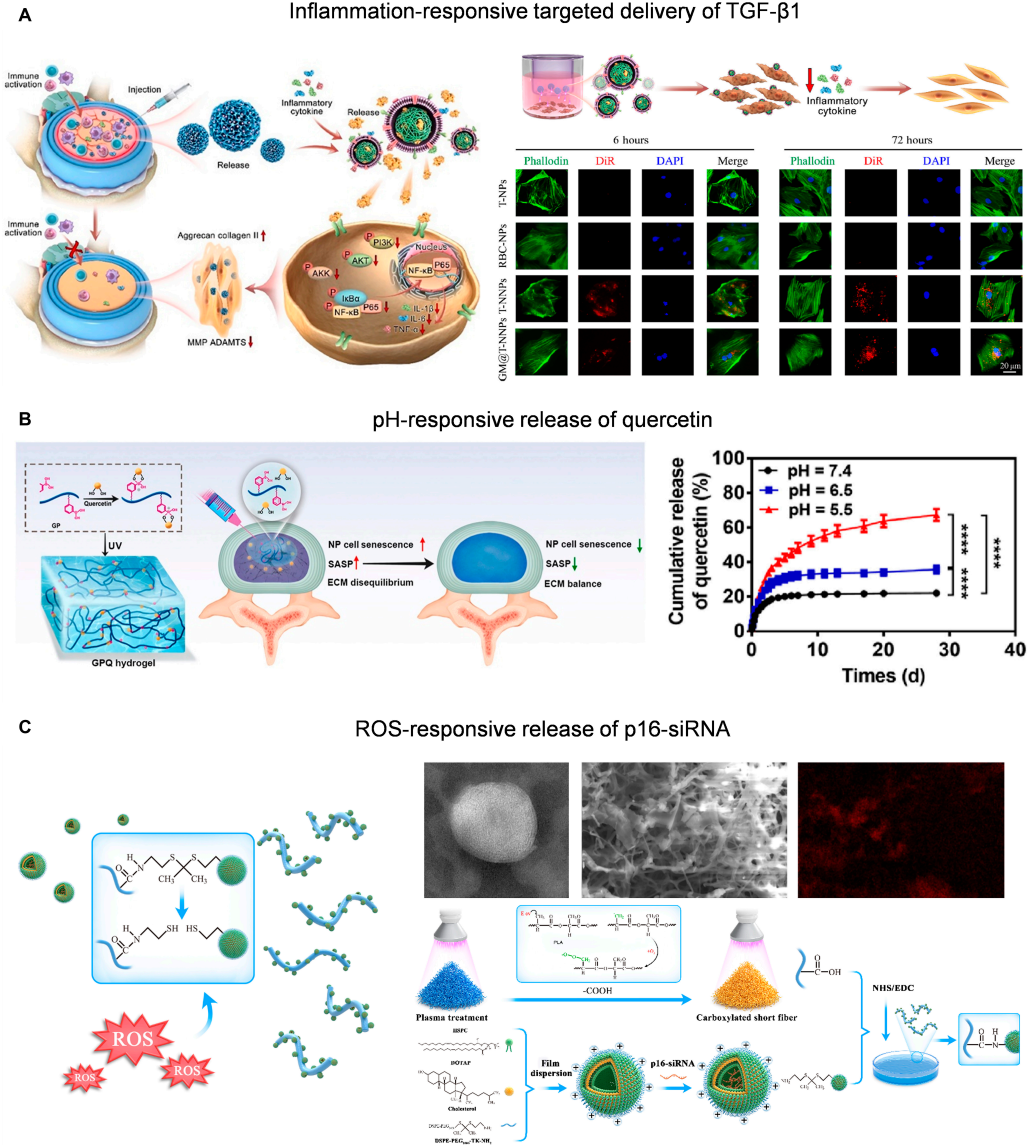
(A) Neutrophil membrane-coated nanoparticles target inflammatory sites for controlled TGF-β1 delivery and IVD regulation. Reproduced with permission [123]. Copyright 2024, Elsevier. (B) pH-responsive hydrogel for targeted drug release in IDD therapy. Reproduced with permission [124]. Copyright 2024, Elsevier. (C) ROS-responsive short fibers release p16–small interfering RNA (siRNA) through thioketal bond cleavage to reverse NP collagen aging. Reproduced with permission [125]. Copyright 2025, Wiley.

In IDD, abnormally growing nerve fibers often cause neurogenic inflammation and further shape the highly inflammatory, hypoxic, acidic, and oxidative microenvironment [67]. This pathological state provides multi-dimensional stimulation signal sources for responsive functional materials, demonstrating the potential in nerve-mediated IDD repair. Neutrophils exhibit a rapid response and chemotactic ability to inflammatory signals, and their membrane structure is an ideal basis for constructing inflammatory microenvironment-responsive nanodelivery systems, endowing materials with specific recognition and regulation of pathological inflammation [122]. Based on this, studies have constructed poly(lactic-co-glycolic acid) (PLGA) nanoparticles coated with neutrophil membrane to deliver transforming growth factor-β1 (TGF-β1), combining inflammation recognition with sustained release. This system alleviated local inflammation and improved ECM homeostasis by inhibiting the phosphatidylinositol 3-kinase (PI3K)–AKT pathway, demonstrating good potential for IVD repair (Fig. 7A) [123]. In addition, another study developed a phenolic boric acid-modified hydrogel that uses the reversible pH response bond formed by phenolic boric acid and quercetin to achieve the intelligent release of quercetin in an acidic environment. This system targeted clear senescent NP cells and alleviated IDD (Fig. 7B) [124]. Similarly, thioketone-modified liposomes achieve self-release in reactive oxygen species (ROS)-enriched degenerative IVD and activate the TGF-β pathway to inhibit NP collagen aging and promote ECM remodeling (Fig. 7C) [125].

**Fig. 8.**
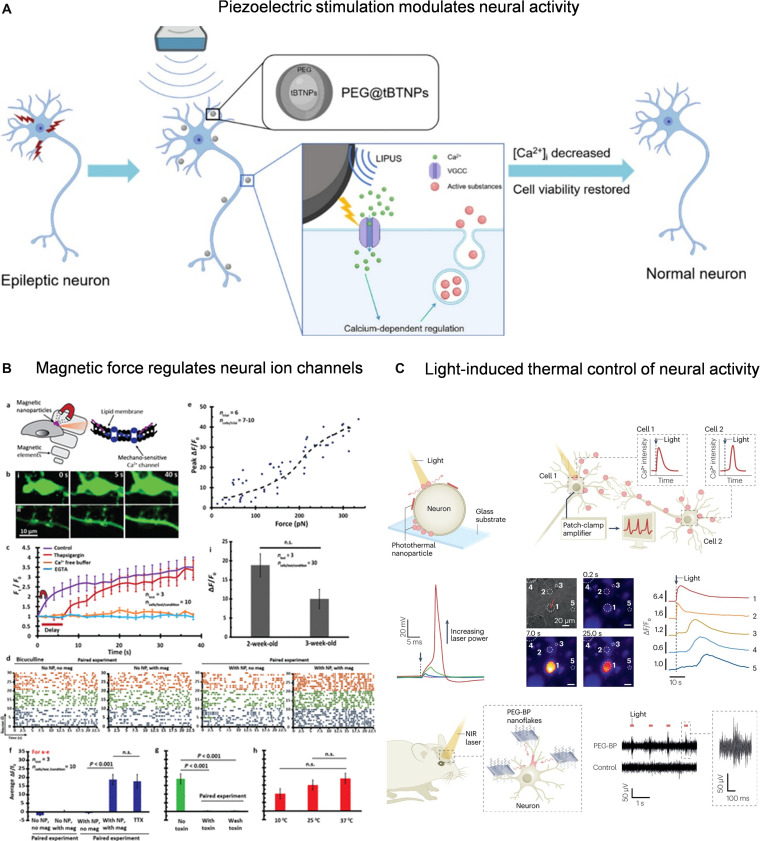
(A) Barium titanate nanoparticle ultrasound-driven electricity generation inhibits neural activity. Reproduced with permission [128]. Copyright 2024, American Chemical Society. (B) Magnetic nanoparticles activate mechanosensitive calcium channels to regulate neural activity. Reproduced with permission [130]. Copyright 2017, American Chemical Society. (C) Photothermal nanomaterials activate thermosensitive channels to achieve neural regulation. Reproduced with permission [131]. Copyright 2023, Springer.

In addition to endogenous signals, functional materials based on electrical, magnetic, and photothermal responses can achieve precise local stimulation under external physical fields. This has become an important strategy for regulating neural excitability and is particularly suitable for intervening in abnormal neural excitability in IDD. For electrical regulation, cathodic electrical stimulation can activate voltage-gated potassium channels and enhance K^+^ efflux, triggering membrane hyperpolarization, thereby increasing action potential threshold and reducing neuronal excitability [126,127]. In addition to the direct electric field effects, electrically responsive materials can achieve indirect neural regulation. For example, piezoelectric materials can generate local electrical signals under an external pressure to simulate electrophysiological stimuli. Studies have shown that polyethylene glycol (PEG)-modified piezoelectric barium titanate nanoparticles can effectively inhibit abnormal neuronal discharge under ultrasound, providing new ideas for the use of piezoelectric materials in neural inhibition (Fig. 8A) [128]. For magnetic regulation, static magnetic field stimulation can regulate the excited state of the nervous system. Related studies have shown that cross-spinal static magnetic fields can reduce the excitability of the cortical spinal tract, demonstrating their potential as neuroregulatory tools [129]. For example, a micropatterned magnetic field platform can remotely activate magnetic nanoparticles, regulate the mechanical sensitivity of Ca²^+^ channels, and alleviate abnormal neural excitation (Fig. 8B) [130]. Photothermal regulation materials rely on local thermal effects induced by near-infrared light, which can alter the electrophysiological properties of neural cell membranes or activate thermosensitive ion channels, thereby achieving precise nongenetic regulation of neural activity (Fig. 8C) [131]. As a photothermal transduction material, polydopamine nanoparticles have excellent biocompatibility and thermal conversion efficiency and can effectively inhibit neuronal discharge under low-dose near-infrared light irradiation [132].

#### Neural invasion inhibition materials based on topology, stiffness, and biochemical barriers

**Fig. 9.**
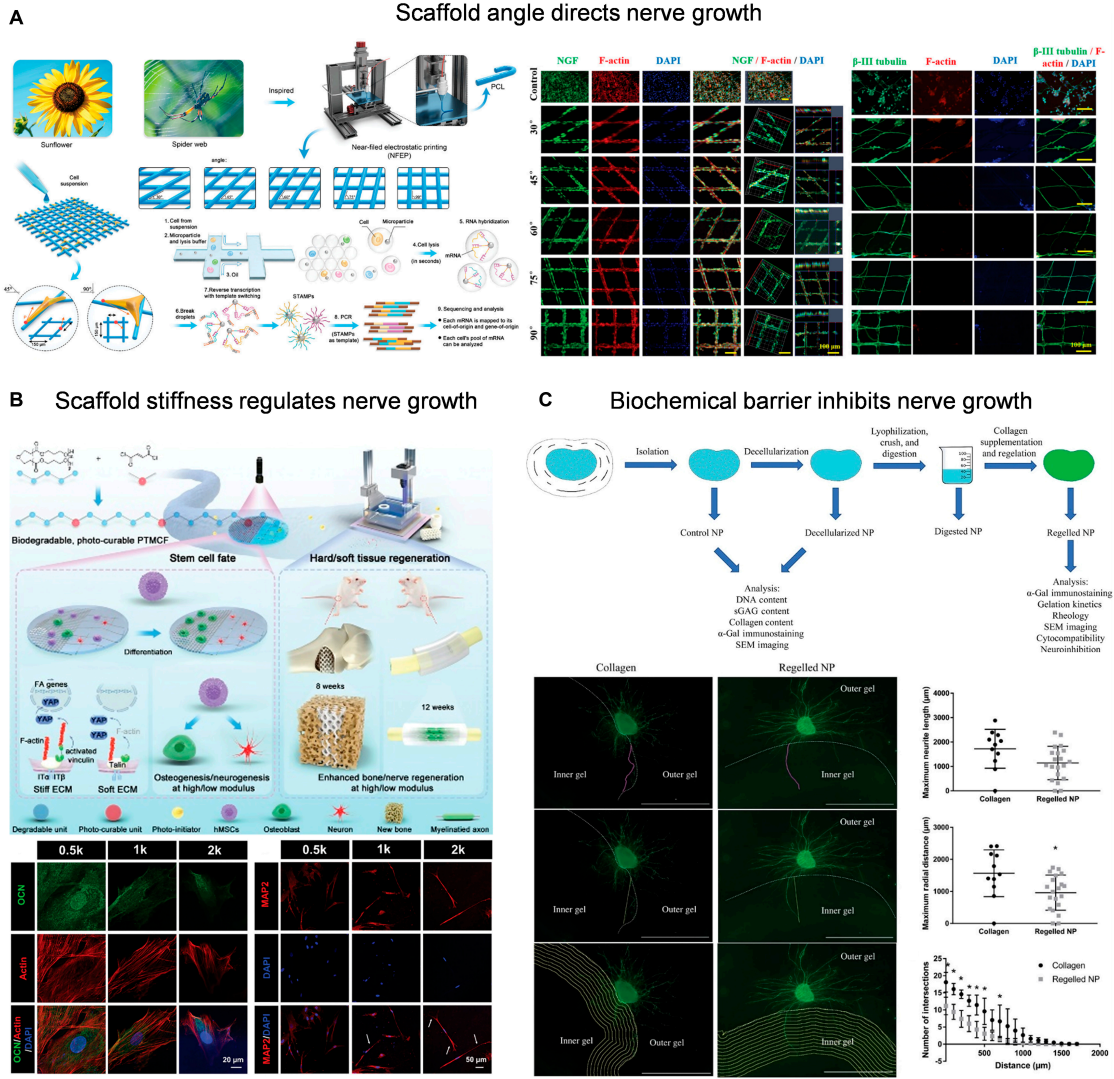
(A) Scaffold angle directs nerve growth. Reproduced with permission [135]. Copyright 2023, Wiley. (B) The stiffness of scaffold regulates nerve growth. Reproduced with permission [137]. Copyright 2024, Wiley. (C) Injectable decellularized NP hydrogel retains sulfated glycosaminoglycans (sGAGs) to suppress nerve ingrowth. Reproduced with permission [140]. Copyright 2022, Wiley.

Recent studies have revealed that physical properties, such as the topological structure [133] and mechanical stiffness [134] of materials, can accurately regulate the growth mode and invasion trend of nerve fibers at the microscopic level by affecting cell adhesion, migration, and differentiation. Rationally designed materials with spatial barrier effects and mechanical rejection ability may effectively inhibit the pathological growth of abnormal nerve fibers in tissues with highly structured IVD and complex microenvironments. For example, 3-dimensional structures with specific geometric angles can activate the miR-222-5p/cbfb/Runx2 key signaling pathway and regulate the differentiation fate of bone marrow mesenchymal stem cells. This markedly enhances the expression of osteogenesis-related genes, promotes bone regeneration and construction of neural vascular networks, and provides new ideas for neural invasion control (Fig. 9A) [135]. Similarly, multilevel biomimetic topological structure can accurately guide and restrict the growth path of axons, reflecting the key role of the microstructure in regulating the direction, speed, and targeting of nerve growth [136].

Sensory axons prefer a soft environment, while a rigid matrix inhibits nerve differentiation and axon extension through mechanical induction pathways, such as YAP and Rho, providing a new idea of physical barriers for inhibiting nerve invasion (Fig. 9B) [137]. For example, studies show that increasing the content of type I collagen in a methacryloylated hyaluronic acid/laminin/collagen ternary hydrogel can markedly improve matrix stiffness and inhibit the length of DRG axon extension in rats, without affecting the number of supporting cells. This suggests that the stiffness of the basement has a negative regulatory effect on sensory nerve axon invasion [138]. Furthermore, chondroitin sulfate proteoglycans in sulfated glycosaminoglycans (sGAGs) have a bidirectional regulatory effect on nerves, which can activate the RhoA/ROCK signaling pathway by binding their chondroitin sulfate side chains to cell membrane receptors. This induces neuronal growth cone collapse and axonal retraction, and concentration-dependent inhibition of sensory nerve axon extension, such as DRG, thus forming a biochemical barrier to prevent nerve invasion [139]. Based on this, researchers developed an acellular injectable hydrogel from pig NP tissue, which retained most of the sGAGs and showed pronounced nerve growth inhibition in the DRG coculture system, suggesting effective blockage of pathological nerve fiber invasion via the sGAG-mediated biochemical barrier mechanism (Fig. 9C) [140]. In addition, some studies introduced enzymes that degrade inhibitory glycosaminoglycans into hydrogels to reshape the scar microenvironment after injury, thereby effectively promoting the regeneration and extension of central nerve axons [141]. This strategy suggests that designing biomaterials that preserve or enhance glycosaminoglycan expression could be used to construct biochemical barriers that inhibit abnormal nerve fiber invasion. This provides new regulatory tools for low-density tissues such as IDD. By integrating spatial configurations, mechanical properties, and biochemical signals, biomaterials can construct microenvironmental barriers with neural exclusion capabilities, thereby becoming an important direction for IDD intervention.

#### Neuroimmune microenvironment regulating materials

**Fig. 10.**
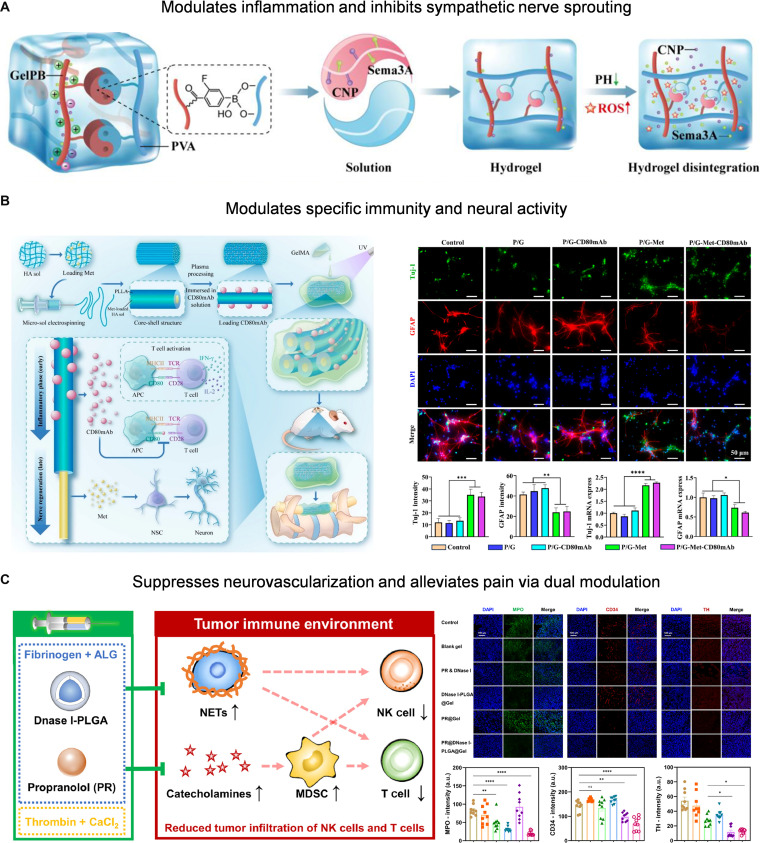
(A) Hydrogel loaded with CNP and Sema3A simultaneously regulates immune polarization and sympathetic innervation. Reproduced with permission [142]. Copyright 2025, Elsevier. (B) “Inner–outer” molecule-loaded fiber-hydrogel scaffold enables coordinated neural and immune modulation. Reproduced with permission [143]. Copyright 2024, American Chemical Society. (C) Hydrogels disrupt neutrophil extracellular trap (NET) and neurotransmitter signaling for dual immune–neural modulation. Reproduced with permission [144]. Copyright 2024, Elsevier.

Interrupting the neural–immune interaction axis requires constructing a biomaterial system with dual regulatory capabilities. Existing cases are mostly from other disease models; however, they still provide important insights into IDD material design. For example, researchers constructed an injectable hydrogel loaded with C-type natriuretic peptide (CNP) and Sema3A, which realized the coordinated intervention of local immune microenvironment regulation and inhibition of excessive sympathetic nerve regeneration in the myocardial infarction model. This markedly reduced macrophage infiltration and promoted M2 polarization while effectively blocking the germination of abnormal sympathetic nerve fibers (Fig. 10A) [142]. Similarly, in the spinal cord injury model, the fiber hydrogel scaffold loaded with metformin and anti-CD80 monoclonal antibody improved the immune microenvironment by inhibiting the T cell costimulatory signal and inducing the recruitment and differentiation of neural stem cells, realizing the dual regulation of the immune and nervous systems (Fig. 10B) [143]. Furthermore, a crosslinked hydrogel loaded with a neutrophil extracellular trap (NET) inhibitor and propranolol can achieve dual inhibition of immune microenvironment remodeling and pathological neurotransmitter activity by degrading NETs and antagonizing catecholamine signaling (Fig. 10C) [144]. These cross-organizational neural immune dual target material strategies provide useful references for interrupting the abnormal neural immune interaction axis in IDD repair. Also, it plays an in-depth role in the intervention and regulation of the “neuro-IVD microenvironment interaction” from the perspective of material means (Table 2).

**Table 2.** Different neural regulatory biomaterials for IVD repair

Category	Biomaterials	Design core	Ref.
Nanomaterial delivery system	TI-cNP-DAF	Delivery of TrkA-IN-1 to suppress inflammation and nerve growth	[119]
	MnO_2_@TMNP	Delivery of MnO_2_ and TrkA inhibition to regulate immune microenvironment and suppress nerve growth	[120]
	miR@A-MSN-MG1	Delivery of miR-26a-5p reduces activation and inflammation of microglia	[121]
Stimulus-responsive functional materials	Inflammatory-responsive	Neutrophil membrane-coated nanoparticles preferentially accumulate in inflammatory regions	[123]
	PH-responsive	PBA forms reversible boronic ester bond with quercetin	[124]
	ROS-responsive	Thioketone bond cleavage in ROS environment	[125]
	Electricity-responsive	Barium titanate piezoelectric nanoparticles inhibit neural activity	[128]
	magnetism-responsive	Magnetic nanoparticles regulate the mechanical sensitivity of Ca²^+^ channels	[130]
	Light-responsive	PDA nanoparticles as photothermal transduction materials inhibit neuronal discharge	[132]
Materials that inhibit neural invasion	Special topological structure materials	Special angle fibers regulate stem cell differentiation; multi-level biomimetic topology structure for precise guidance and restriction of axonal growth pathways	[135,136]
	High stiffness material	Enhancing matrix stiffness to suppress axonal elongation	[138]
	Biochemical barrier material	Increase the content of sGAGs in NP	[140,141]
	GelPB/PVA hydrogel loaded with CNP/Sema3A	Reduce macrophage infiltration and promote M2 polarization while effectively blocking abnormal sympathetic nerve fiber sprouting	[142]
Neuroimmune microenvironment regulating materials	P/G-Met-CD80 mAb scaffolds	Inhibiting T cell costimulatory signals improves the immune microenvironment and induces recruitment and differentiation of neural stem cells	[143]
	PR@DNase I-PLGA@Gel	By degrading NETs and antagonizing catecholamine signaling, dual inhibition of immune microenvironment remodeling and pathological neurotransmitter activity is achieved	[144]

ROS, reactive oxygen species

## Outlook and Discussion

Neural regulation strategies provide new treatment ideas for IDD, and regenerative medicine materials provide highly flexible intervention carriers; however, current research remains largely confined to early stages of “mechanism exploration and functional verification”, and a systematic breakthrough in accurate identification and intervention of “neuro-IVD microenvironment interactions” has not yet been achieved. In the absence of a deep understanding of the mechanism of neural–immune mechanical interactions, it is difficult to balance the dual needs of biological regulation and structural repair, resulting in insufficient clinical transferability, limited intervention persistence, and targeting.

### The neural regulatory mechanism has not been fully analyzed yet

Previous studies have revealed changes in the expression of neurotrophic factors in IDD and the promoting effect of abnormal nerve fiber growth on pain and matrix degradation; however, there remains a lack of integrated models for the spatiotemporal dynamic behavior of neurons and glial cells at different stages of degeneration. Signal coupling between axon-guiding molecules (such as Semaphorin and Netrin) and local immune cells (macrophages and satellite glial cells), and how central peripheral neuroinflammatory feedback systematically forms and amplifies the pathological process of IDD, remains underexplored. Leveraging high-throughput omics and in vivo imaging, future research should aim to construct a multilevel neural immune mechanical interaction network map covering molecules, cells, and tissues. Such frameworks will clarify key nodes and modifiable targets at different stages to provide a solid theoretical basis for precise neural regulation strategies. In particular, neuropeptides such as CGRP exhibit dual roles in physiological and pathological states, which highlights that future interventions should prioritize “precise regulation” over “one-way inhibition”. Notably, CGRP may exert stage-dependent regulatory effects during IDD progression, reflecting its dual roles in inflammation and tissue repair. The dynamic modeling of “neuro-IVD microenvironment interaction” will be essential in revealing the mechanisms of degeneration progression and neural sensitization patterns.

### The material regulation strategy still lacks personalized and clinical translation considerations

Currently, the vast majority of neural regulatory biomaterials have been validated only in small animal models, overlooking the substantial differences in size, proteoglycan content, mechanical gradient, microenvironment acidity, and alkalinity of human IVD. In addition, interspecies variations in neuro-immune interactions may further complicate translational extrapolation, underscoring the need for preclinical models that more closely mimic human IVD physiology and immune–neural crosstalk. Although these materials have shown potential in small animal experiments, clinical translation faces many challenges, including long-term safety, degradation product impact, individualized design, and reproducibility that still need to be addressed. Particularly, the biocompatibility and degradation behavior of these materials under the harsh IVD microenvironment remain to be further clarified. Moreover, the release kinetics, biocompatibility, and long-term safety of the in vivo degradation products of material drug delivery systems have not been fully evaluated. Customized designs based on patient imaging characteristics, molecular spectra, and pain phenotypes are largely absent. In addition, critical aspects such as dose-timing optimization, targeted delivery, and reproducible manufacturing process standards for clinical applications remain underexplored. Furthermore, more systematic in vivo validation is required, and future work could benefit from comparative analyses summarizing the translational outcomes of different material systems. To bridge these gaps, it is imperative to integrate materials science with clinical needs, perform data-driven optimization based on large-scale samples, and conduct early human trials to achieve stable and safe therapeutic effects. Future translation will also benefit from patient-specific designs guided by imaging and pain phenotypes, as well as attention to scalability, sterilization, and regulatory pathways for neural regulatory biomaterials. In the process of clinical translation, it is also necessary to focus on potential immune reactions and biocompatibility issues, and avoid them through strategies such as material surface modification, sustained release control, and multiple rounds of in vitro/in vivo safety assessments.

### Multidisciplinary integration

Neural regulation and repair of IDD span fields such as biomaterials science, neuroscience, tissue engineering, immunology, computational biology, and rehabilitation medicine, necessitating the creation of a truly interdisciplinary collaboration platform. Future research should integrate spatial omics and high-resolution in vivo imaging technology to monitor the material neural immune mechanical interaction process in real time. In addition, machine learning approaches should be leveraged to predict the impact of material formulations on neural behavior and tissue repair. These design results should be incorporated into early clinical rehabilitation pathways to optimize intervention plans. Such integration will accelerate the clinical implementation of neuroregulatory biomaterials and ultimately achieve precise intervention in the “neuro-IVD microenvironment interaction” across scales and dimensions.

## Data Availability

All data included in this study are available upon request by contact with the corresponding authors.
